# Dehydroepiandrosterone alleviates hypoxia‐induced learning and memory dysfunction by maintaining synaptic homeostasis

**DOI:** 10.1111/cns.13869

**Published:** 2022-06-15

**Authors:** Ruili Guan, Changhao Yang, Jianbin Zhang, Jianyu Wang, Rui Chen, Peng Su

**Affiliations:** ^1^ Key Laboratory of Environmental Medicine Engineering, Ministry of Education, School of Public Health Southeast University Nanjing China; ^2^ Medical School of Chinese PLA Chinese PLA General Hospital Beijing China; ^3^ Department of Occupational & Environmental Health and the Ministry of Education Key Lab of Hazard Assessment and Control in Special Operational Environment School of Public Health, Fourth Military Medical University Xi'an China

**Keywords:** dehydroepiandrosterone, hippocampus, hypoxia, neuron, synaptic function

## Abstract

**Aims:**

Hypoxia causes plenty of pathologies in the central nervous system (CNS) including impairment of cognitive and memory function. Dehydroepiandrosterone (DHEA) has been proved to have therapeutic effects on CNS injuries by maintaining the homeostasis of synapses, yet its effect on hypoxia‐induced CNS damage remains unknown.

**Methods:**

In vivo and in vitro models were established. Concentrations of glutamate and γ GABA were tested by ELISA. Levels of synapse‐associated proteins were measured by western blotting. Density of dendritic protrusions of hippocampal neurons was assessed by Golgi staining. Immunofluorescence was adopted to observe the morphology of primary neurons. The novel object recognition test (NORT) and shuttle box test were used to evaluate cognition.

**Results:**

Dehydroepiandrosterone reversed abnormal elevation of glutamate levels, shortenings of neuronal processes, decreases in the density of dendritic protrusions, downregulation of synaptosome‐associated protein (SNAP25), and impaired cognition caused by hypoxia. Hypoxia also resulted in notably downregulation of syntaxin 1A (Stx‐1A). Overexpression of Stx‐1A dramatically attenuated hypoxia‐induced elevation of glutamate. Treatment with DHEA reversed the Stx‐1A downregulation caused by hypoxic exposure.

**Conclusion:**

Dehydroepiandrosterone may exert a protective effect on hypoxia‐induced memory impairment by maintaining synaptic homeostasis. These findings offer a novel understanding of the therapeutic effect of DHEA on hypoxia‐induced cognitive dysfunction.

## Graphical abstract

In vivo and in vitro data revealed that DHEA alleviated hypoxia‐induced learning and memory dysfunction via maintaining synaptic homeostasis. Furthermore, DHEA may exert protective effect by targeting Stx‐1A.

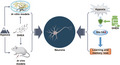

## 1 INTRODUCTION

Hypoxia is an environmentally stressful state, also it is a major factor affecting human life activities in the plateau environment.^1^ It has already been confirmed by studies that human tissues and organs are devastated by hypoxia; in particular, the central nervous system (CNS) is damaged by hypoxia.^2^, ^3^, ^4^ Brain tissues are characterized by high metabolism and high energy consumption, and thus are extremely sensitive to hypoxia. Studies have reported that hypoxia treatment induces peripheral and CNS inflammation, leading to neuronal death and neurocognitive deficits.^5^, ^6^ Other studies have shown that the damage of hypoxia on neuronal function may be due to impairment of synaptic function. In vitro evidences have revealed that exposure to hypoxia alternated the level of glutamic acid decarboxylase enzymes and affected synapse stability.^7^ Through observing the alternation of synapses directly, it has been disclosed that being exposed to hypoxia results in the decline of synaptic density between spinal cord motoneuron and descending dopaminergic axons.^8^ Thus, how to prevent CNS from hypoxia injuries is the hot spot in the neuroscientific field and environmental scientific field. Previous studies have shown that medications, such as acetazolamide, melatonin, etc., may play roles in alleviating neurological damage caused by hypoxia, but their protective effects still need further validation.^9^ In the present studies, we found that DHEA exerted protective effects on hypoxia‐induced neural impairments.

Dehydroepiandrosterone (DHEA), a precursor substance of sex hormones, is mainly synthesized and secreted by the adrenal cortex. DHEA is also synthesized directly in neurons and astrocytes. It has been confirmed that the central nervous system can be effectively protected by DHEA. In vivo evidence showed that mice with contusive spinal cord damage recovered better in locomotor functions after treatment with DHEA.^10^ Furthermore, it has been proved in vitro that treatment of DHEA attenuated toxicity of colchicine, NMDA, 1‐methyl‐4‐phenylpyridine (MPP+), and glutamate on rat cerebellar granule cells.^11^ DHEA promotes both presynaptic actions (releasing of norepinephrine, acetylcholine, and glutamate) and postsynaptic actions (inhibition of voltage‐gated calcium currents, agonism of N‐methyl‐D‐aspartate [NMDA] receptor, and GABAergic antagonism).^12^, ^13^, ^14^ Nevertheless, what effect DHEA exerts on the damage of neurons caused by hypoxia has not yet been proved. In the present studies, we found that DHEA had a beneficial role in neural function in response to hypoxia treatment.

Studies have shown that multiple mechanisms are involved in hypoxia‐induced neural damage.^15^, ^16^ The targets at which the development of synapses is changed by hypoxia still remain unclear. It has been recently disclosed that a membrane protein named syntaxin 1A (Stx‐1A) plays an important role in the maintenance of the normal function of synapse. It concerns the release of neurotransmitters and the docking of synaptic vesicles. Thereby, Stx‐1A serves neurotransmission crucially. Some studies have reported that Stx‐1A might play a protective role in cardiomyocytes under ischemic and hypoxia condition.^17^, ^18^ Studies also revealed that Stx‐1A might contribute to the onset of neurodevelopmental disorders such as children ADHD^19^ and Asperger syndrome.^20^ Combining with these findings, we assumed that Stx‐1A may exert a potential role in hypoxia injuries of CNS.

Our study showed that DHEA alleviates hypoxia‐induced memory impairment and reverses the abnormal elevation of neuronal glutamate production, and it may exert a protective effect by targeting Stx‐1A. These results provide novel insights into an effective solution to neurological damage induced by hypoxia.

## 2 METHODS

### 2.1 Animals and treatments

Forty grown‐up male C57BL/6J mice (weight 18 ~ 22 g) were obtained from the Air Force Medical University Animal Center. Mice were divided randomly according to the experiment plan and were raised under regulated lighting conditions (12 h/12 h light/dark cycle), temperature (25 ± 2°C), and humidity (40 ~ 60%). In the current study, DHEA was dissolved in corn oil and intraperitoneally injected following a dose of 25 and 50 mg/kg every 48 h. After the acclimation period, we randomized the mice into 4 groups (*n* = 6 in each group): Con (control), Hyp (hypoxia), Hyp + 25 mg/kg (hypoxia + 25 mg/kg DHEA), and Hyp + 50 mg/kg (hypoxia + 50 mg/kg DHEA). The control group was kept under the normal condition as described above, whereas the other three groups were exposed to a hypoxic condition equal to an altitude of 6000 m by raising in a hypoxia cubage (Fenglei Co. Ltd.) for 14 days continuously according to our former report.^21^ Mice were treated with continuous DHEA injection for 2 weeks simultaneous with exposure to hypoxia. The animals were sacrificed for further analysis after 1 week of behavioral testing. Whole brains were used for Golgi stain. The hippocampus tissues were dissected closely with the use of tiny forceps. Hippocampus tissues were used for immunoblotting analysis and measurements of glutamate and γ GABA levels. All procedures were carried out strictly according to the international standards of animal care guidelines.^22^ Our experimental procedures were approved by the Air Force Medical University Institutional Care and Use Committee (Permit Number: SCXK‐2019‐0007).

### 2.2 Cell culture and exposure to hypoxia

C57BL/6J pup mouse hippocampal primary neurons were cultured. Once the surface vascular membrane was entirely removed, brains were dissected with a pipette in HBSS (Gibco, 1582478) and then separated into individual cells. After centrifuging for 2 min at the rate of 150 ~ 250 *g* and discarding the supernatant, cell pellets were suspended by tender pipette in Neural Basal Medium (Gibco), which contained 2 mM GlutaMAX (Gibco), B‐27® (Invitrogen), 12.5 μM l‐glutamic acid (Sigma), and 1% penicillin‐streptomycin (Gibco). The separated primary cells were inoculated at the density of 8000 cells/cm^2^ on a coverslip coated with poly d‐lysine and were cultured 37°C with 5% CO_2_.

HT22 cells (mouse hippocampal neuronal cell line) were purchased from American Type Culture Collection (ATCC, USA). The Dulbecco's modified Eagle's medium (DMEM) (Gibco) added with 10% fetal bovine serum (FBS, Gibco) was used to culture HT22 cells. Cells were cultured at 37°C with 5% CO_2_.

HT22 cells and primary hippocampal neurons were divided into four groups: Con (control), Hyp (hypoxia), Con + 20 μM DHEA (control + 20 μM DHEA), and Hyp + 20 M DHEA (hypoxia + 20 μM DHEA). A microaerophilic incubation system (Don Whitley) filled with 1% oxygen and 5% carbon dioxide was used to build up hypoxic exposure models in vitro as described previously.^21^ The culture medium was pretreated in the microaerophilic incubation system for 9 h before use.

### 2.3 CCK‐8 analysis

The CCK8 cell counting kit‐8 reagent produced by the USA ApexBio Technology was used to determine how cell viability was affected by the corresponding treatment. The kit uses a water‐soluble tetrazolium salt to quantify the number of live cells by producing an orange formazan dye upon bioreduction in the presence of an electron carrier. HT22 cells were incubated for 1 day (24 h) in a 96‐well plate, each well containing 1 × 10^4^ cells, and treated correspondingly with DHEA. Following the exposure, 10 μl of CCK8 reagent was supplemented and cells were then incubated for 2 h at 37°C. In the end, a microplate reader (Tecan; Spark) was used to figure out the value of optical density (OD) at 450 nm. Three independent experiments were performed. We calculated cell viability as follows: (the OD value of experimental groups ‐ the OD value of blank groups)/(the OD value of control groups ‐ the OD value of blank groups).

### 2.4 Western blot

Lysis buffer that contains protease inhibitors was used to extract proteins from C57BL/6J mouse brain cells, and western blotting was employed to detect the protein expression. To be detailed, the detection kit of BCA‐200 (Thermo) was used to quantify the proteins. 20 mg of the protein sample was then treated with sodium dodecyl sulfate‐polyacrylamide gel electrophoresis. Protein was further transferred on a PVDF membrane, which was then kept for 2 h in TRIS‐buffered saline (Sigma) that contains 5% skimmed milk powder. The membrane was incubated overnight with primary antibodies at 4°C. Horseradish peroxidase‐conjugated antibodies were incubated for 2 h at 25°C. Chemiluminescence system (BioRad) was employed to visualize protein bands. After being normalized by β‐actin, image J was adopted to evaluate the protein expression.

### 2.5 ELISA

In order to conduct the γ GABA measurement, both cell culture lysates and brain tissue were treated with the RIPA buffer. Following the instructions, mouse γ‐aminobutyric acid enzyme‐linked immunosorbent assay kit (Jiancheng Bioengineering Institute, China) was employed to measure the level of GABA in concentration‐determined samples.

### 2.6 Measurement of glutamate levels

Hippocampus tissues were accurately weighted, normal saline was supplemented 9 times the tissue weight (9 ml/g). Tissues were homogenized mechanically under an ice bath and then were centrifuged. 10% supernatant was taken, and 0.6 ml of detection reagent was added. After centrifuging, 0.5 ml of supernatant was taken for testing. Working fluids were prepared following the instructions (Jiancheng Bioengineering Institute, China): 37°C water bathed for 40 min; the absorbance of each tube was measured at 340 nm wavelength.

### 2.7 Immunofluorescent staining

Cells cultured on the glass coverslip were washed with PBS and were fixed with 4% paraformaldehyde. Then, cells on coverslips were permeabilized at 4°C by using 0.25% Triton X‐100 (MP Biomedicals, 9002‐93‐1). Next, coverslips were blocked by 5% heat shock bovine albumin (MP Biomedicals, FC0077). Coverslips were incubated overnight at 4°C with primary antibodies (MAP2). Then, coverslips were incubated with a secondary antibody. Coverslips were fixed on the glass slide and then visualized with a confocal laser scanning microscope (Nikon).

### 2.8 Golgi staining of neuronal dendrites

According to the manufacturer's instruction, FD Rapid GolgiStainTM Kit (FD NeuroTechnologies) was used to carry out the staining procedure. The dipping Solution A/B was prepared in advance at least 24 h before being used. Brain tissues were soaked in the dipping solution A/B and kept in the dark for 2 weeks at room temperature. A/B solution was replaced the next day. Then, tissues were transferred to solution C and kept for 3 days in the dark. 24 h later, solution C was replaced. 150 μm sections were cut with a slide section machine. In the end, Solution C was used to fix sections on gelatin‐coated slides.

The dipping Solution D/E was prepared. Sections were rinsed in double distilled water, then sections were kept for 10 min in solution D/E. After being rinsed for another time, sections were dehydrated by sequentially being rinsed with 50%, 75%, and 95% ethanol. Sections were then cleared three times in xylene and were coverslipped. With a bright‐field microscope, they were then imaged. Golgi‐stained sections were kept in the dark at room temperature.

### 2.9 Shuttle box assay

The shuttle box was a rectangular box divided into two compartments, which were connected by a hole. 12 electrified iron rods were installed on the bottom of the box. Lights were attached to the top of each box. After acclimatizing to the environment, mice were sequentially put into any box, then the door was closed. 3 min later, the light on one side was turned on, and electric rods under the dark box were electrified simultaneously. The mice were then forced to run to the light side under the stimulation of electric current. During this period, the behavior of each subject was recorded by the TSE software. After each mouse finished the experiment, the box was wiped with 75% alcohol to remove the odor. The experiment was repeated for four consecutive days. Finally, the numbers of active avoidances completed per day by each mouse were calculated to evaluate the learning and memory abilities.

### 2.10 Novel object recognition test

We follow the previous procedure for the novel object recognition test (NORT) 20. After acclimatizing to the environment, mice were sequentially put in an open field size of which is 48 × 34 × 18 mm. Before the test, we placed two same objects named, respectively, A1 and A2 in the field equidistant from the corner of the enclosure. Then mice were permitted to explore freely in the open field for 3 min. On the next day, before 3 min of exploration, object A1 was substituted by object B with a completely different shape. Familicidal object exploration time (TA) and novel object exploration time (TB) were recorded on the video tracking system (DigiBehave system, Xinruan), and the discriminant index (DI) [DI = (TB − TA) / (TB + TA)] were calculated. The open field area was cleaned carefully by using 70% ethanol before the next test to eliminate the effect of residual odor prompts.

### 2.11 In vitro lentivirus infection

HT 22 cells were seeded at a density of 4.5 × 10^4^ cells/cm^2^. The medium was changed after culturing for 24 h, and the virus solution was added following the dose of MOI = 10. 24 h later, the medium was changed to a fresh solution.

### 2.12 In vitro siRNA interference

HT22 cells were at a density of 4.5 × 10^4^ cells/cm^2^. Calculate the required volume of small interference working fluid. Take a 6 cm dish as an example: 500 μl Opti‐MEM medium and 20 μl siRNA/NC were added to prepare solution A; 500 μl Opti‐MEM medium was added with 10 μl LP 2000 to prepare solution B. The liquids were mixed and left at room temperature for 5 min. The above two liquids A and B were then mixed thoroughly to prepare a working solution and were incubated at room temperature for 20 min. After removal of the culture medium, 2 ml of Opti‐MEM and 1 ml of interference working solution were added. After incubation for 6 h, the working solution was replaced by the normal cell culture medium.

### 2.13 Statistical analysis

All data acquired were analyzed by SPSS 20.0 software (IBM Co.). Data were presented as mean ± standard error of the mean (SEM). Depending on the normality of the variables, ANOVA and Friedman analysis were performed when comparing more than two groups in an experiment, and *t*‐tests and Mann–Whitney *U* tests were used to compare the differences between the two groups. Probability values (*p* values) lower than 0.05 were considered statistically significant.

## 3 RESULTS

### 3.1 Hypoxic exposure results in aberrant viability and neuronal dysfunction in HT22 cells

**FIGURE 1:**
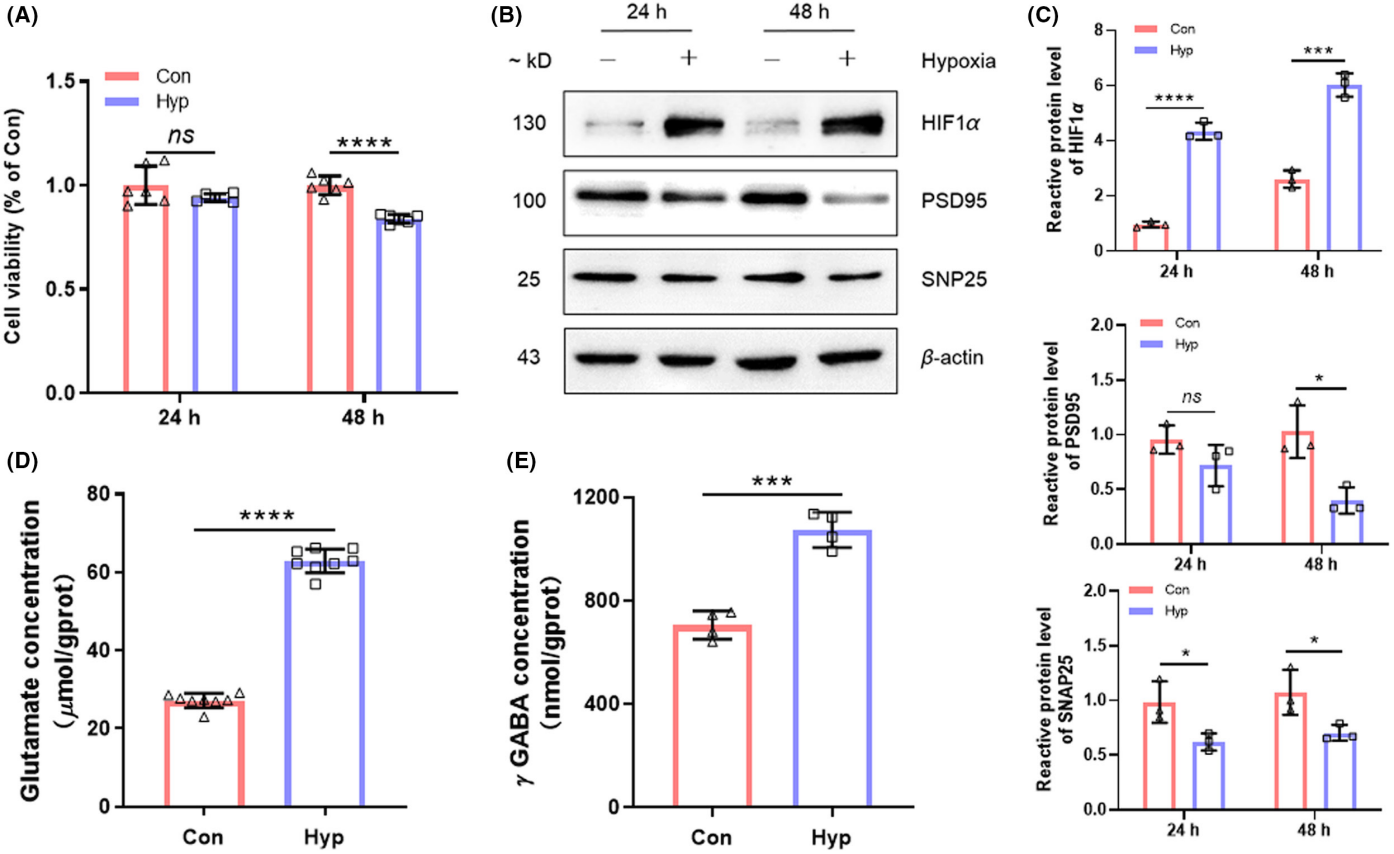
Hypoxic exposure results in aberrant viability and neuronal dysfunction in HT22 cells. (A) Viability of HT22 cells was tested by CCK8 after hypoxic exposure for 24 and 48 h (*n* = 5, mean ± SEM). (B) Protein levels (HIF1α, PSD95, and SNAP25) were measured by western blotting after hypoxic exposure. (C) Gray intensity analysis of HIF1α, PSD95, and SNAP25 (*n* = 3, mean ± SEM). β‐actin was used as internal reference. (D) Glutamate concentrations were tested after hypoxic exposure (*n* = 3, mean ± SEM). (E) γ GABA concentrations were tested after hypoxic exposure (*n* = 3, mean ± SEM). ns, no significant; *p < 0.05, **p < 0.01, and ****p < 0.0001

To clarify the appropriate duration of hypoxic exposure, we performed CCK8 analysis on HT22 cells exposed to hypoxia (1% O_2_) for 24 and 48 h (Figure 1A). CCK8 analysis is to detect the cell viability of each group. After being exposed to hypoxia for 48 h, the viability of HT22 cells decreased significantly (*p* < 0.0001). Western blot data of HT22 cells showed increases in HIF1 α protein level and decreases in PSD95 and SNAP25 protein levels under hypoxic status for 48 h (*p* < 0.05, Figure 1B, C). In 24 h hypoxia group, only HIF1 α and SNAP25 changed notably (*p* < 0.05), while PSD95 did not decrease significantly (*p* > 0.05). To further explore the synaptic function, we measured the content of glutamate and γ GABA in HT22 cells exposed to hypoxia for 48 h (Figure 1D,E). Both glutamate and γ GABA elevated significantly in HT22 cells after hypoxic exposure (*p* < 0.001).

### 3.2 DHEA reverses neuronal function impaired by hypoxic exposure

**FIGURE 2:**
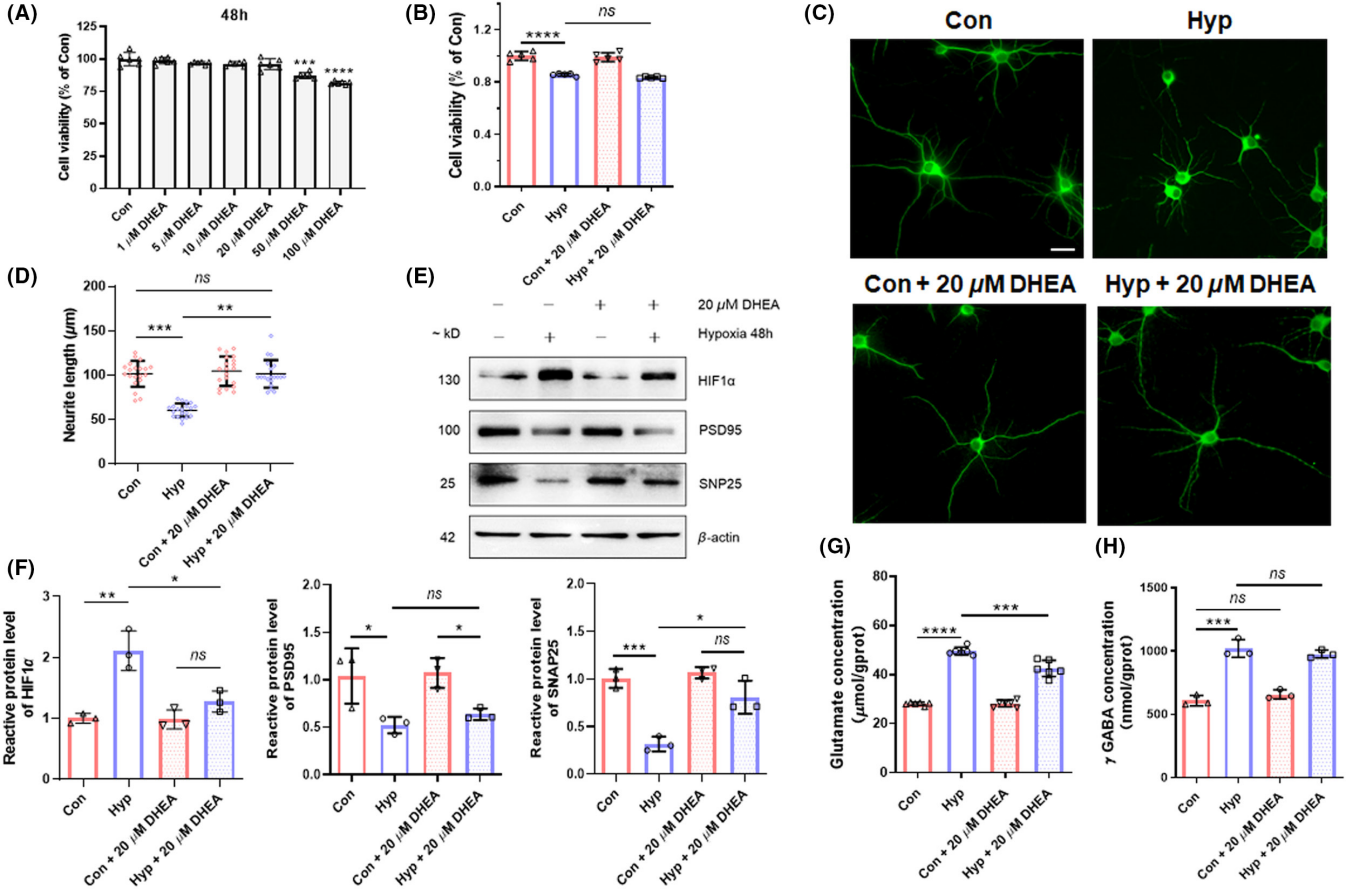
DHEA reverses neuronal function impaired by hypoxic exposure. (A) Representative viability of HT22 cells was tested by CCK8 after treatment of DHEA in different concentrations (*n* = 5, mean ± SEM). (B) Representative cell viability of HT22 cells tested by CCK8 following hypoxic exposure with or without DHEA (*n* = 5, mean ± SEM). (C) Effects of DHEA on cell morphology in hypoxia‐exposed mice primary‐cultured hippocampal neurons (scale bar = 20 μm). (D) Neurite length of primary‐cultured hippocampal neurons after hypoxic exposure with or without treatment of DHEA (*n* = 5, mean ± SEM). (E) Protein level of HIF1α, PSD95, and SNAP25 in HT22 cells measured by western blotting after hypoxic exposure with or without DHEA. (F) Gray intensity analysis of HIF1α, PSD95, and SNAP25 (*n* = 3, mean ± SEM). (G) Glutamate concentration of HT22 cells tested after hypoxic exposure with or without DHEA (*n* = 3, mean ± SEM). (H) γ GABA concentration of HT22 cells tested after hypoxic exposure with or without DHEA (*n* = 3, mean ± SEM). ns, no significant; *p < 0.05, **p < 0.01, ***p < 0.001, and ****p < 0.0001

To explore the influence of DHEA in different concentrations on the viability of HT22 cells, CCK8 analysis revealed that treatment of DHEA for 48 h in 1 ~ 20 μM did not affect cell viability, while 50 and 100 μM of DHEA significantly decreased viability of HT22 cells (*p* < 0.01 compared with the control group, Figure 2A). Hence, we chose 20 μM as the appropriate treatment concentration for DHEA. To determine whether the observed decreases in cell viability caused by hypoxia were ameliorated by DHEA, we evaluated the viability of HT22 cells under hypoxia conditions with or without treatment of DHEA (Figure 2B). No significant difference was found between the hypoxia group and hypoxia with the 20 μM DHEA group (*p* > 0.05). The effects of DHEA on neurite outgrowth were examined in hypoxia‐exposed mice with primary hippocampal neurons. Hypoxic exposure significantly inhibited the length of neurites by nearly 40% (*p* < 0.001, Figure 2C,D). After treatment with DHEA, the length of neuronal neurites recovered to a level close to that of the control group (*p* < 0.01 compared with hypoxia group, *p* > 0.05 compared with control group). To assess the effects of DHEA on synaptic function impaired by hypoxia, western blot data revealed that the protein level of SNAP25 was significantly reversed with treatment of DHEA, upregulation of HIF1α was also notably reversed (*p* < 0.05). However, downregulation of PSD95 induced by hypoxia showed no distinction after treatment of DHEA (*p* > 0.05, Figure 2E,F). Abnormally elevated glutamate concentration in HT22 cells after hypoxic exposure was significantly reversed with treatment of DHEA (*p* < 0.001, Figure 2G), while increases in γ GABA showed no significant change (*p* > 0.05, Figure 2H).

### 3.3 DHEA reverses mice memory function impairment caused by hypoxic exposure

**FIGURE 3:**
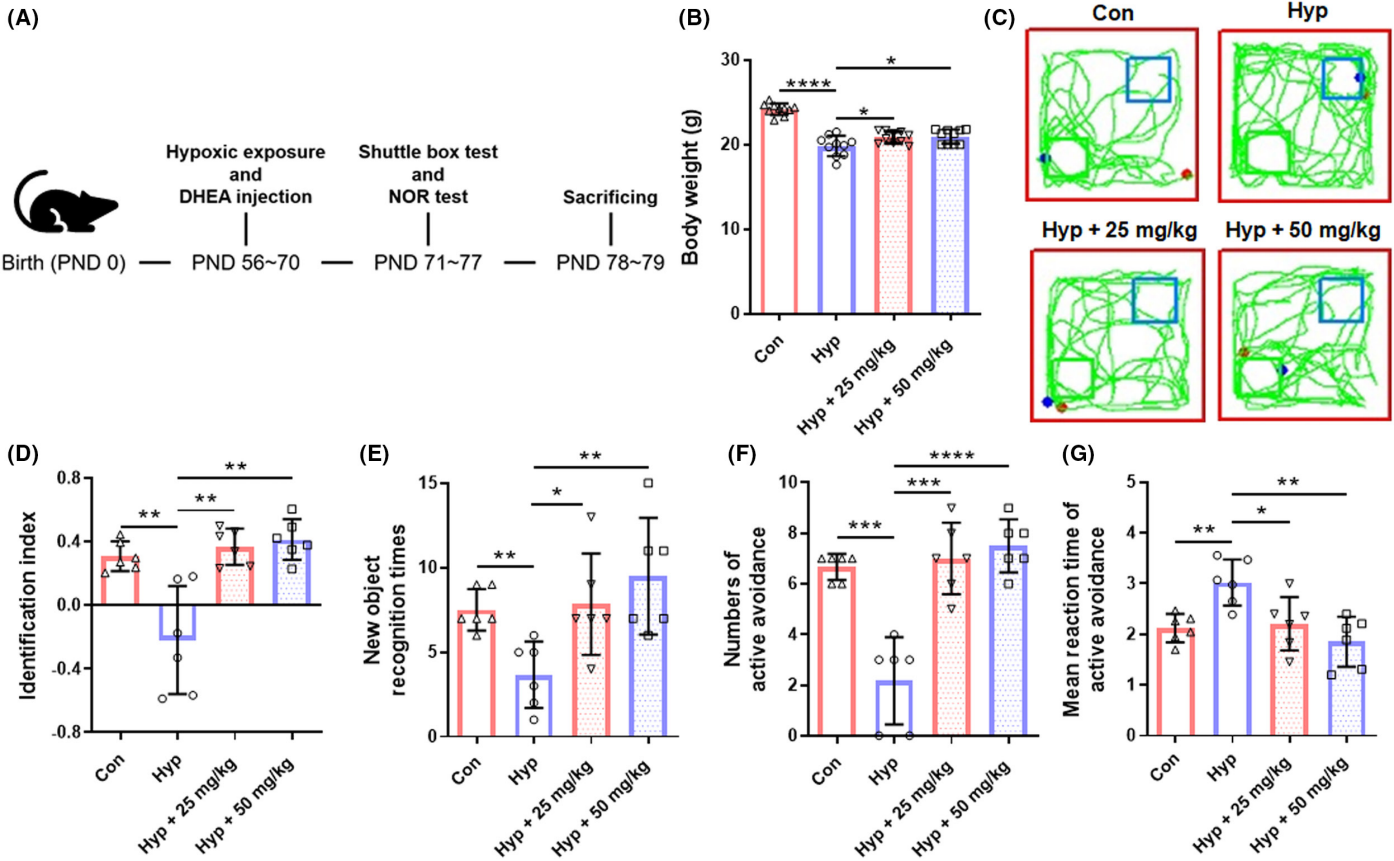
DHEA reverses mice memory function impairment caused by hypoxic exposure. (A) The experimental process proposal. (B) Body weight of mice after 2 weeks of hypoxic exposure (*n* = 10, mean ± SEM). (C) Representative locomotion tracking routes of mice exposed to hypoxia in NORT with or without DHEA. (D) Representative identification index of mice exposed to hypoxia for 2 weeks in NORT (*n* = 6, mean ± SEM). (E) Representative time of recognition mice used on new objects in the NORT test following 2 weeks of hypoxic exposure with or without DHEA (*n* = 6, mean ± SEM). (F) Representative active avoidance numbers of mice in shuttle box assay following 2 weeks of hypoxic exposure with or without DHEA (*n* = 6, mean ± SEM). (G) Representative mean reaction times of active avoidance of mice in shuttle box assay following 2 weeks of hypoxic exposure with or without DHEA (*n* = 6, mean ± SEM). *p < 0.05, **p < 0.01, ***p < 0.001, and ****p < 0.0001

Grouping and treatments of animals were shown in Figure 3A. Mice in each group were weighed after hypoxic exposure. Data showed that weights were notably reduced after hypoxic exposure (*p* < 0.0001), and DHEA treatment significantly reversed weight loss caused by hypoxia (*p* < 0.05 compared with hypoxia group, Figure 3B). To appraise the influence of DHEA on mice's cognitive function and memory function after being exposed to hypoxia, NORT, and shuttle box assay were performed. NORT data revealed that the hypoxia group spends more time than the control group to explore a novel object (*p* < 0.01). Surprisingly, both identification index and recognition times in the hypoxia group were completely reversed to normal levels after treatment with DHEA (*p* < 0.05 compared with hypoxia group, Figure 3C–E). The shuttle box test showed that the quantity of mice in the hypoxic group to avoid electric shock was notably shortened compared with the control group (*p* < 0.001, Figure 3F). The mean reaction time of the active avoidance was also significantly extended in the hypoxia group (*p* < 0.01, Figure 3G). With the treatment of DHEA, the impairment of working memory induced by hypoxia was totally reversed (*p* < 0.05 compared with the hypoxia group, Figure 3F,G).

### 3.4 DHEA reverses impairment of dendritic plasticity and synaptic function in mice hippocampus induced by hypoxic exposure

**FIGURE 4:**
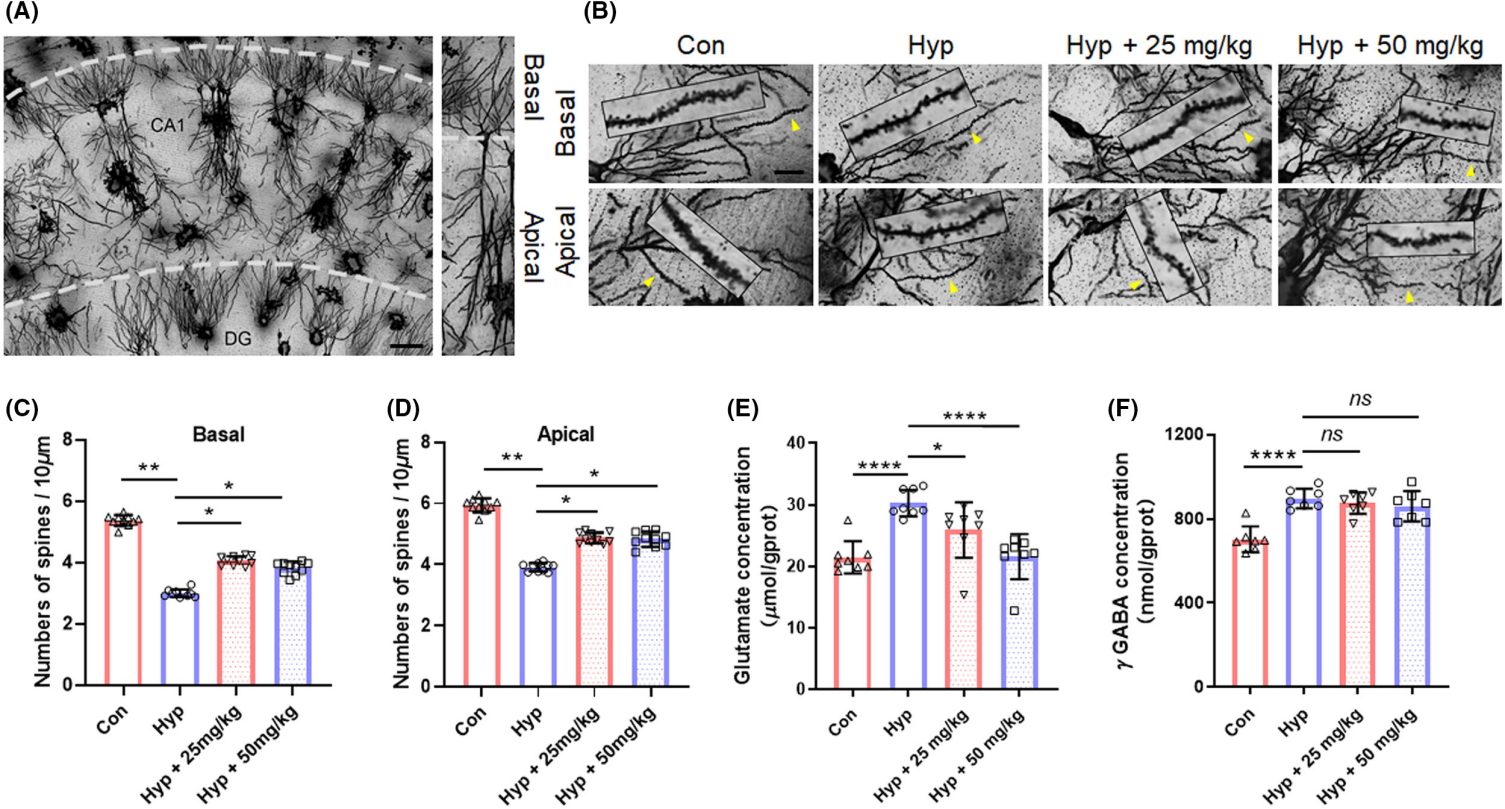
DHEA reverses impairment of dendritic plasticity and synaptic function in mice hippocampus induced by hypoxic exposure. (A) Left: Neurons in the hippocampus through Golgi staining (scale bar = 50 μm). DG = dentate gyrus. Right: Pyramidal neurons in CA1 through Golgi staining. (B) Basal and apical dendritic spines located in CA1 region of hippocampus (scale bar = 10 μm). (C, D) Statistical analysis of hypoxic exposure's influence on basal (C) and apical spines (D). Data are presented as mean ± SEM of per 10 μm spines in each group (32 neurons/16 mice for all groups). (E) Glutamate concentration of hippocampus tissues tested after hypoxic exposure with or without DHEA (*n* = 3, mean ± SEM). (F) γ GABA concentration of hippocampus tissues tested after hypoxic exposure with or without DHEA (*n* = 3, mean ± SEM). ns, no significant; *p < 0.05, **p < 0.01, and ****p < 0.0001

As the structural basis of memory and learning functions, dendritic plasticity has been proved to play a vital role in hypoxia‐induced cognitive deficits.^21^ First, Golgi Staining was used to identify the dendritic plasticity. Locations of the CA1 region in hippocampus and distributions of basal and apical spines in pyramidal neurons were shown in Figure 4A. In CA1 region, the exposure to hypoxia led to a notable decrease in dendritic spine density of apical dendrites and basal dendrites of pyramidal neurons. Injection of DHEA with doses of 25 and 50 mg/kg significantly reversed the decreases in dendritic spine density (*p* < 0.05, Figure 4B–D). Abnormally ascended glutamate concentration in mice hippocampus after hypoxic exposure was significantly reversed with treatment of DHEA (*p* < 0.05, Figure 4E), while increases in γ GABA displayed no significant alternation (*p* > 0.05, Figure 4F).

### 3.5 DHEA reverses hypoxia‐induced downregulation of Stx‐1A

**FIGURE 5:**
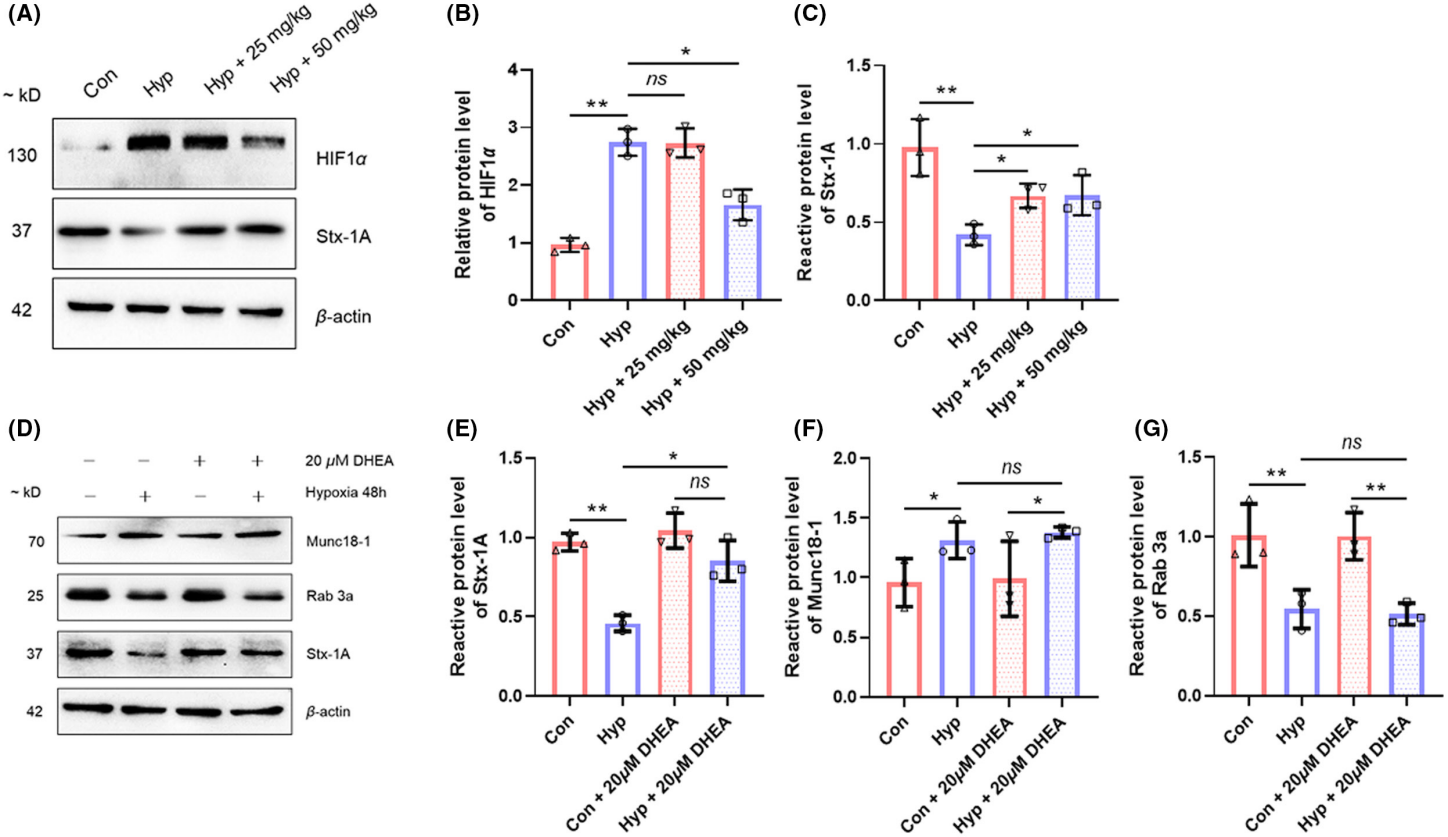
DHEA reverses hypoxia‐induced downregulation of Stx‐1A. (A) Protein level of HIF1α and Stx‐1A in mice hippocampus tissues measured by western blotting after hypoxic exposure with or without DHEA. (B) Gray intensity analysis of HIF1α and Stx‐1A (*n* = 3, mean ± SEM). (C) Protein level of Stx‐1A, Munc18‐1, and Rab 3a in HT22 cells measured by western blotting after hypoxic exposure with or without DHEA. (D–G) Gray intensity analysis of Stx‐1A, Munc18‐1, and Rab 3a (*n* = 3, mean ± SEM). β‐actin was used as internal reference. ns, no significant; *p < 0.05 and **p < 0.01

To elucidate the mechanism underlying the neuronal protective effects of DHEA on hypoxic exposure, we examined the expression of numerous synaptic‐related proteins located in the presynaptic membrane after exposure to hypoxia (Figure S1). Among these proteins, we found that syntaxin 1A (Stx‐1A) significantly downregulated in hypoxic exposed HT22 cells, and downregulation was reversed by DHEA (*p* < 0.05, Figure 5A,C). We used western blot analysis to further assess the effects of DHEA on Stx‐1A after hypoxic exposure in vivo. Consistent with our experiments in vitro, the protein level of Stx‐1A was significantly decreased after hypoxic exposure and was reversed with treatment of DHEA (*p* < 0.05, Figure 5D,E). We also explored changes in other presynaptic membrane proteins including Rab3a and Munc18‐1 in hypoxic exposed HT22 cells after DHEA treatment. Data showed that the protein level of Munc18‐1 increased significantly, and the protein level of Rab3a decreased notably after hypoxic exposure. Both changes were not significant reverse by DHEA treatment (*p* > 0.05, Figure 5F,G). Furthermore, elevated HIF1α protein level induced by hypoxia was also significantly reversed by DHEA (*p* < 0.05, Figure 5A,B).

### 3.6 Stx‐1A is involved in hypoxia‐induced synaptic dysfunction

**FIGURE 6:**
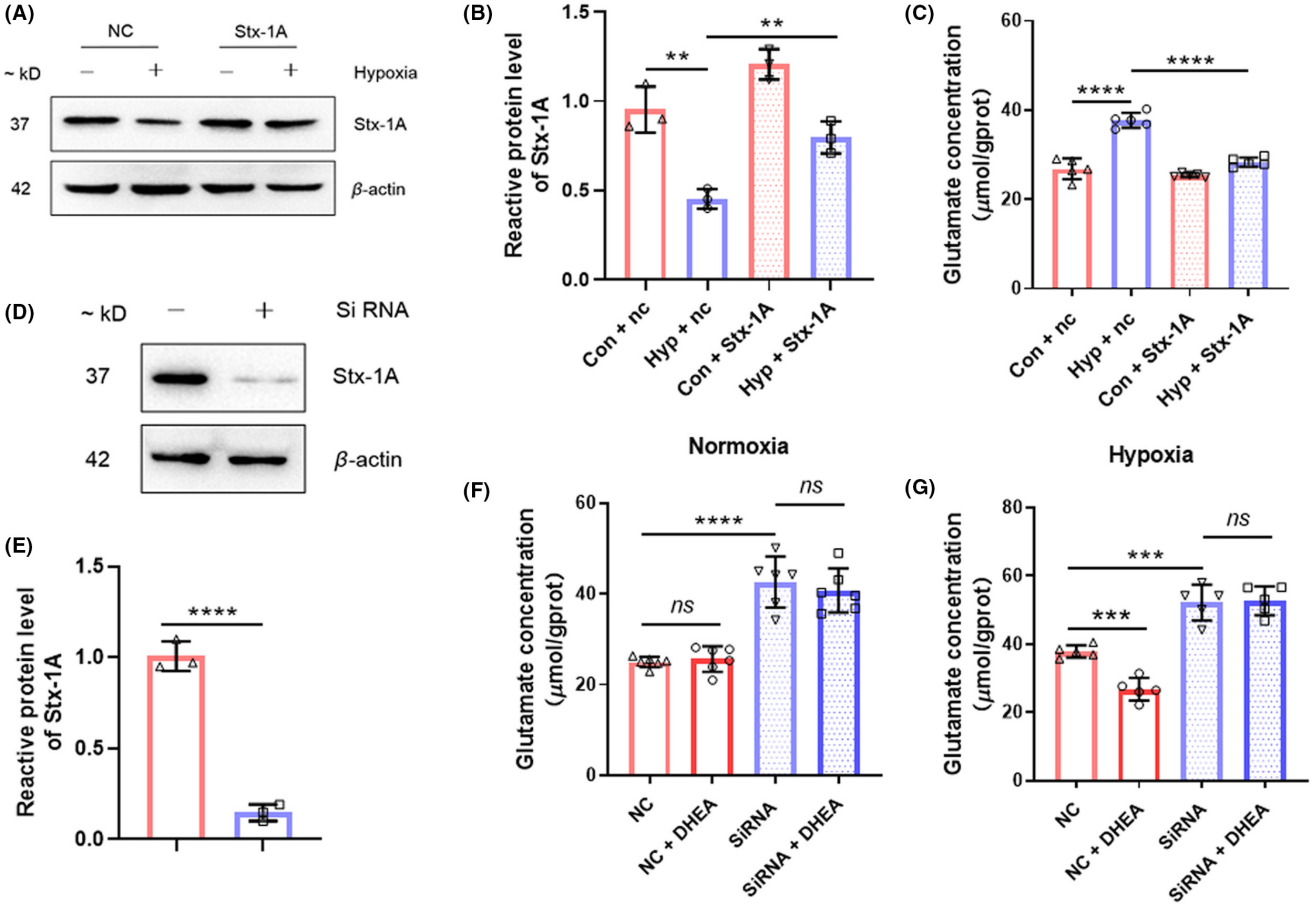
Stx‐1A is involved in hypoxia‐induced synaptic dysfunction. (A) Representative immunoblotting of Stx‐1A in HT22 cells under hypoxic exposure with or without overexpression of Stx‐1A. (B) Gray intensity analysis of Stx‐1A (*n* = 3, mean ± SEM). (C) Glutamate concentration of HT22 cells tested after hypoxic exposure with or without overexpression of Stx‐1A (*n* = 3, mean ± SEM). (D) Representative immunoblotting of Stx‐1A in HT22 cells with or without knockdown of Stx‐1A. (E) Gray intensity analysis of Stx‐1A (*n* = 3, mean ± SEM). (F) Glutamate concentration of HT22 cells tested after normoxia exposure with or without DHEA treatment and knockdown of Stx‐1A (*n* = 3, mean ± SEM). (G) Glutamate concentration of HT22 cells tested after hypoxic exposure with or without DHEA treatment and knockdown of Stx‐1A (*n* = 3, mean ± SEM). ns, no significant; *p < 0.05, **p < 0.01, ***p < 0.001, and ****p < 0.0001

To determine whether Stx‐1A is involved in hypoxia‐induced synaptic dysfunction, overexpression of Stx‐1A was performed in HT22 cells. Our results revealed that the protein level of Stx‐1A was significantly rescued after overexpressed in HT‐22 cells exposed to hypoxia (*p* < 0.01 compared with hypoxia group, Figure 6A,B), indicating that transfection of Stx‐1A with lentivirus was a success. Measurement of glutamate levels showed that abnormally elevated glutamate concentration in HT22 cells after hypoxic exposure was significantly reversed after overexpression of Stx‐1A (*p* < 0.0001 compared with hypoxia group, Figure 6C). To further confirm the changes in glutamate concentration in HT22 cells were related to alterations in the protein level of Stx‐1A, transfection of siRNA was performed. Data showed that protein expression of Stx‐1A was significantly decreased after siRNA transfection (*p* < 0.0001, Figure 6D,E). Measurement of glutamate levels showed that knockdown of Stx‐1A abnormally elevated glutamate concentration in HT22 cells with or without hypoxia exposure (*p* < 0.001), and protective effects of DHEA were completely eliminated by knockdown of Stx‐1A (Figure 6F,G). Therefore, Stx‐1A has involved in the regulation of synaptic function under hypoxia exposed conditions, and the protective effects of DHEA might be mediated by the regulation of Stx‐1A.

## 4 DISCUSSION

The deficit in cognitive function is one of the most important injuries in response to hypoxic exposure in the CNS.^23^ As reported, the exposure to hypoxia results in cognitive dysfunction, memory loss, attention deficit, and even Alzheimer's disease.^24^, ^25^, ^26^ It is known that hippocampus is the center of learning and memory and is highly sensitive to hypoxia and thereby most likely to be damaged in this condition.^27^, ^28^ The current research made exploration into how hypoxic exposure impacted memory, cognitive ability, and synaptic functions in hippocampus. In vitro and in vivo evidences showed that the morphology of neuronal spines was abnormal under the pressure of hypoxia. Besides, the suppression of SNAP25 and PSD95 was also observed. Furthermore, glutamate and γ GABA concentrations elevated significantly after exposure to hypoxia. These findings confirmed the detrimental effects of hypoxia on the impairment of memory functions.

The synthesis of DHEA occurs in the CNS.^14^ Compared with the peripheral nervous system, its concentration in the CNS is much higher,^12^, ^13^, ^14^ which indicates that DHEA may play an important role in the CNS. It has been confirmed that DHEA has the function of adjusting various types of synaptic transmission, including cholinergic,^29^ GABAergic,^30^ dopaminergic, and glutamatergic transmission.^31^, ^32^, ^33^ Studies reported that DHEA levels are closely related to cognitive performance. High levels of DHEA were related to increased memory scores in transient brain ischemia models.^34^ However, whether DHEA can alleviate brain damage under hypoxic conditions remains unclear. Based on this, experiments in vivo and in vitro were adopted in the current study to figure out whether DHEA exerts therapeutic effects on the CNS under hypoxic circumstances. Our data showed that DHEA cannot relieve the decrease in cell viability caused by hypoxia, suggesting that DHEA may protect neurons through other paths. By inhibiting the abnormal increases of glutamate and adjusting the abnormal decrease of SNAP25, DHEA contributes to alleviate the synaptic damage caused by hypoxia. However, the changes in PSD95 and γ GABA were not appreciably improved. These results indicated that under hypoxia conditions, the effect of DHEA on the presynaptic membrane is more pronounced than that of the postsynaptic membrane, suggesting that its target is more likely to be located on the presynaptic membrane of hippocampal neurons.

To confirm the protective role of DHEA in CNS, we conducted in vivo experiments, and our results indicated that DHEA at a dose of 25 and 50 mg/kg had no significant effect on weight loss caused by low oxygen exposure. Results from the shuttle box (SB) test and the novel object recognition test (NORT) showed that DHEA notably reversed the damage to memory and cognitive performance.

Consistent with our results, previous studies also observed retention in the learning function of mice after treatment with DHEA.^14^ It has also been proved in previous studies that DHEA enhanced memory retention, and learning incapability resulting from compounds including dizocilpine, ethanol, anisomycin, and scopolamine can be relieved by DHEA.^35^ Furthermore, the positive effects of DHEA on alleviating cognitive impairment were confirmed by its association with the improved cognitive capability of patients who were suffering from Alzheimer's disease.^13^ These findings suggested that DHEA has potential neuroprotective effects on learning and memory functions.

In line with our in vitro results, the content of glutamate and γ GABA in hippocampus of mice increased significantly after exposure to hypoxia, while DHEA only notably ameliorate the abnormal increase of glutamate. The release of glutamate from exocytotic vesicles is the most abundant exciting neurotransmitter in the human brain. In this process, glutamate is stored and then fused into synaptic vesicles in the presynaptic membrane.^36^ In the glutamate synapse, special postsynaptic structures which are named dendritic spines, are usually associated with the presynaptic terminals. Our Golgi staining results showed that hypoxic exposure significantly reduced the dendritic spine density of hippocampus neurons, while administration of DHEA notably improved dendritic spine density. These results suggested that the beneficial effects of DHEA on hippocampal neurons were exerted by preventing the release of glutamate.

Stx‐1A belongs to the family of syntaxin. Together with SNAP 25 and VAMP (vesicle‐associated membrane protein), Stx‐1A contributes to the form of SNARE (soluble N‐ethylmaleimide sensitive factors attachment protein).^37^ It is involved in the sprouting of neurites and in the growth of axons.^38^ It has been confirmed that Stx‐1A (−/−) mice's memory function was damaged when Stx‐1A was disrupted through gene knockout, which disclosed that Stx‐1A gene is probably in connection with the plasticity of synapses. Besides, several studies showed that Stx‐1A knockout impaired the cognition process.^39^ However, it still remains unclear what role does Stx‐1A play in CNS damage caused by hypoxia. We examined the expression of Stx‐1A in vitro and in vivo models as abovementioned. Excitingly, we found that Stx‐1A was significantly downregulated both in vivo and in vitro, and it was significantly reversed after the administration of DHEA. Overexpression of Stx‐1A significantly reversed the abnormal elevation of glutamate concentration after exposure to hypoxia, which suggested that Stx‐1A is involved in the regulation of synaptic function in hypoxia‐exposed models. After the interference of Stx‐1A, intracellular glutamate content increased significantly, and DHEA treatment could not reverse the accumulation of intracellular glutamate content after normoxia or hypoxia exposure. Therefore, the protective effects of DHEA might be mediated by the regulation of Stx1A.

In summary, our results confirmed that DHEA alleviates hypoxia‐induced learning and memory dysfunction via maintaining synaptic homeostasis. Furthermore, our results suggested that DHEA may exert a protective effect by targeting Stx‐1A. These findings offer a novel understanding of the preventive effect of DHEA on cognitive dysfunction under exposure to hypoxia.

## AUTHOR CONTRIBUTIONS

Peng Su, Jianbin Zhang, and Rui Chen conceived the paper. Changhao Yang and Ruili Guan mainly analyzed the data. Ruili Guan, Changhao Yang, and Jianyu Wang developed the model and performed the molecular biology experiments. Ruili Guan and Jianyu Wang participated in the in vivo experiments. Peng Su and Changhao Yang wrote the paper. All authors read and approved the final manuscript.

## CONFLICT OF INTEREST

The authors declare that the research was conducted in the absence of any commercial or financial relationships that could be construed as a potential conflict of interest.

## Supporting information


Figure S1

## Data Availability

No data are available.
