# Supplementary material for: Dehydroepiandrosterone alleviates hypoxia‐induced learning and memory dysfunction by maintaining synaptic homeostasis
Source: CNS Neurosci Ther. 2022 Jun 15;28(9):1339–50. doi: 10.1111/cns.13869 (PMC9344085; doi:10.1111/cns.13869)
Supplement: Supplementary file 1 — Figure S1 [file CNS-28-1339-s001.pptx]

## Slide 1
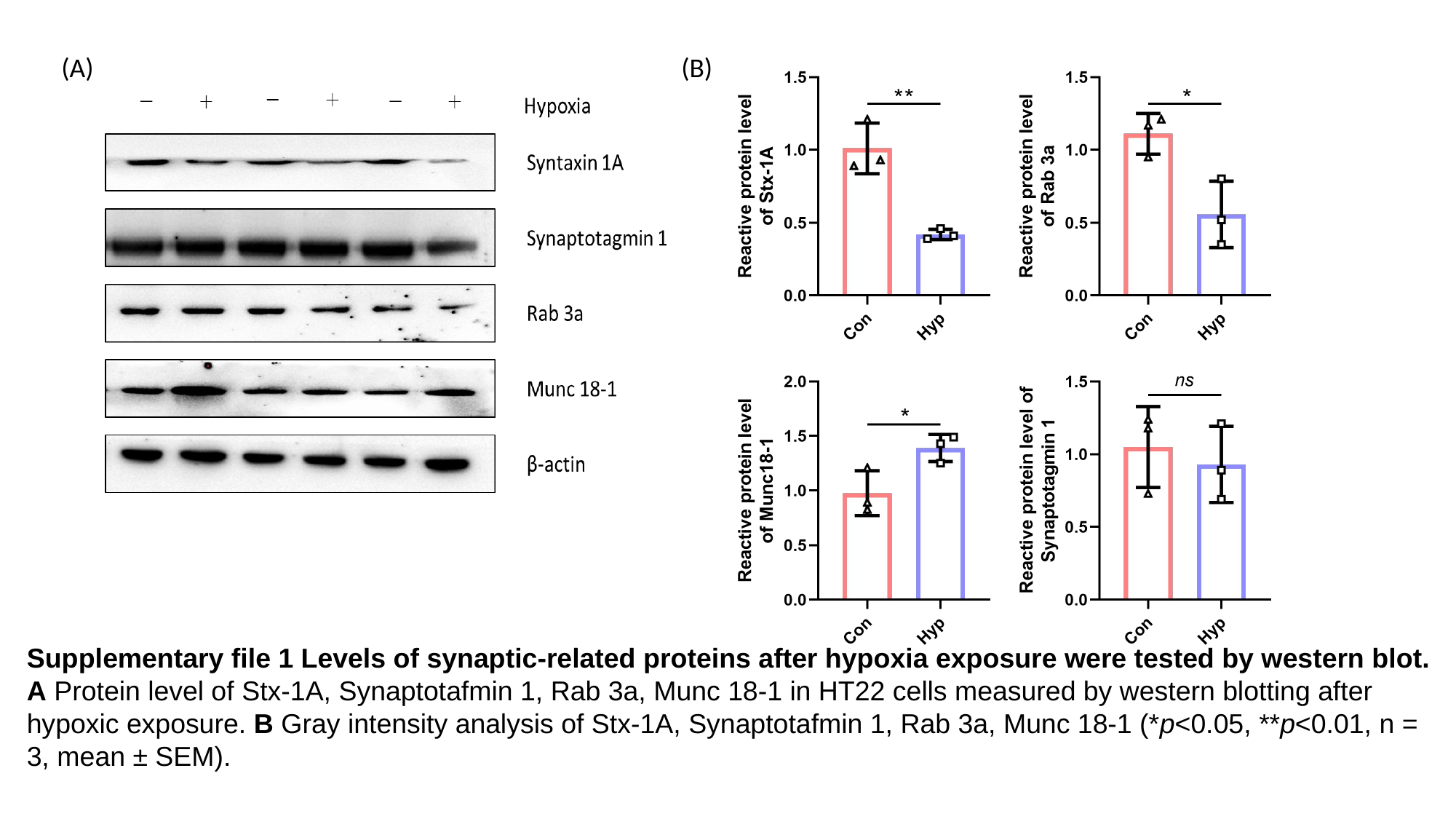

(A)
(B)
Supplementary file 1 Levels of synaptic-related proteins after hypoxia exposure were tested by western blot.
A Protein level of Stx-1A, Synaptotafmin 1, Rab 3a, Munc 18-1 in HT22 cells measured by western blotting after hypoxic exposure. B Gray intensity analysis of Stx-1A, Synaptotafmin 1, Rab 3a, Munc 18-1 (*p<0.05, **p<0.01, n = 3, mean ± SEM).

## Slide 2
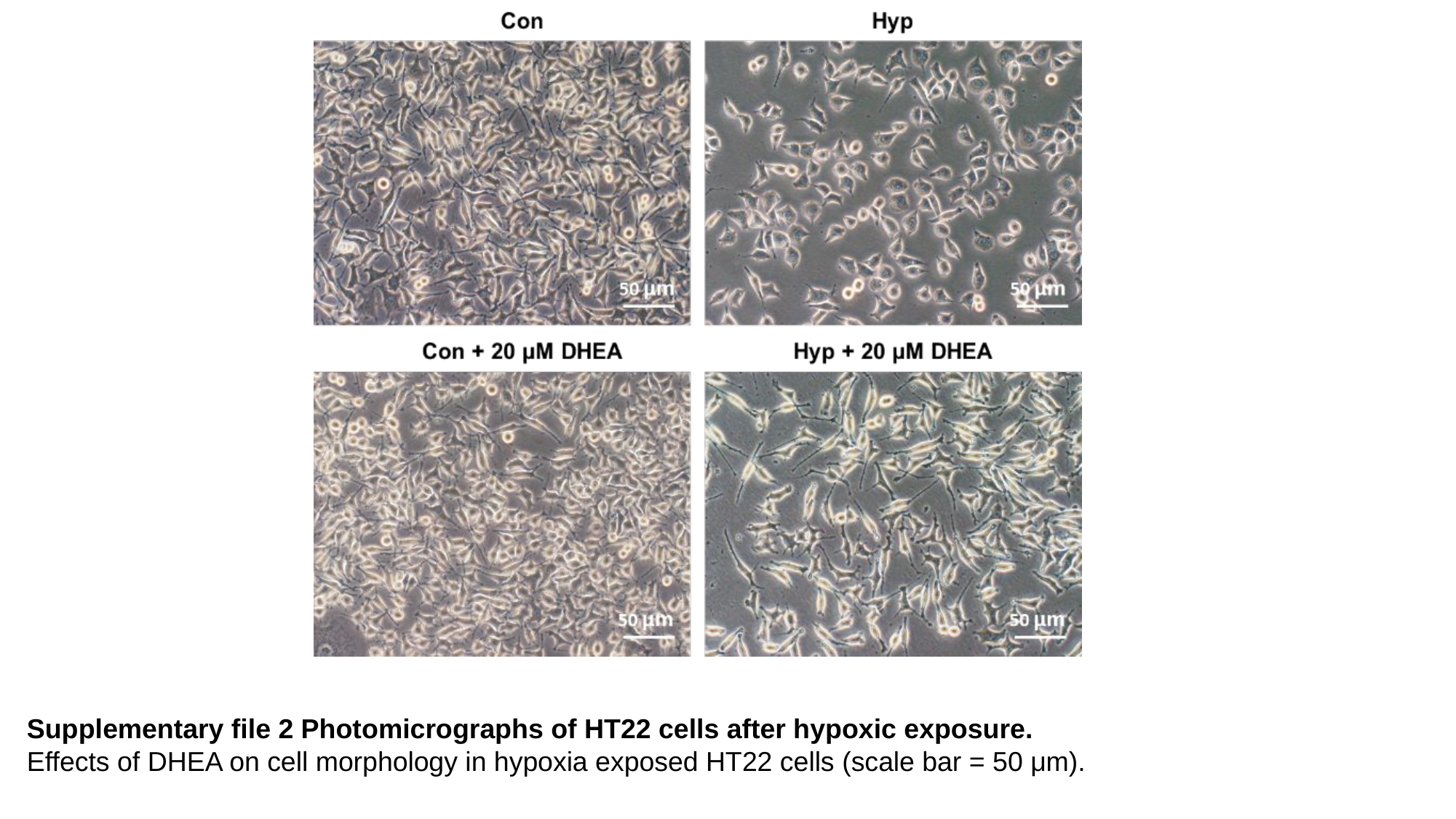

Supplementary file 2 Photomicrographs of HT22 cells after hypoxic exposure.
Effects of DHEA on cell morphology in hypoxia exposed HT22 cells (scale bar = 50 μm).

## Slide 3
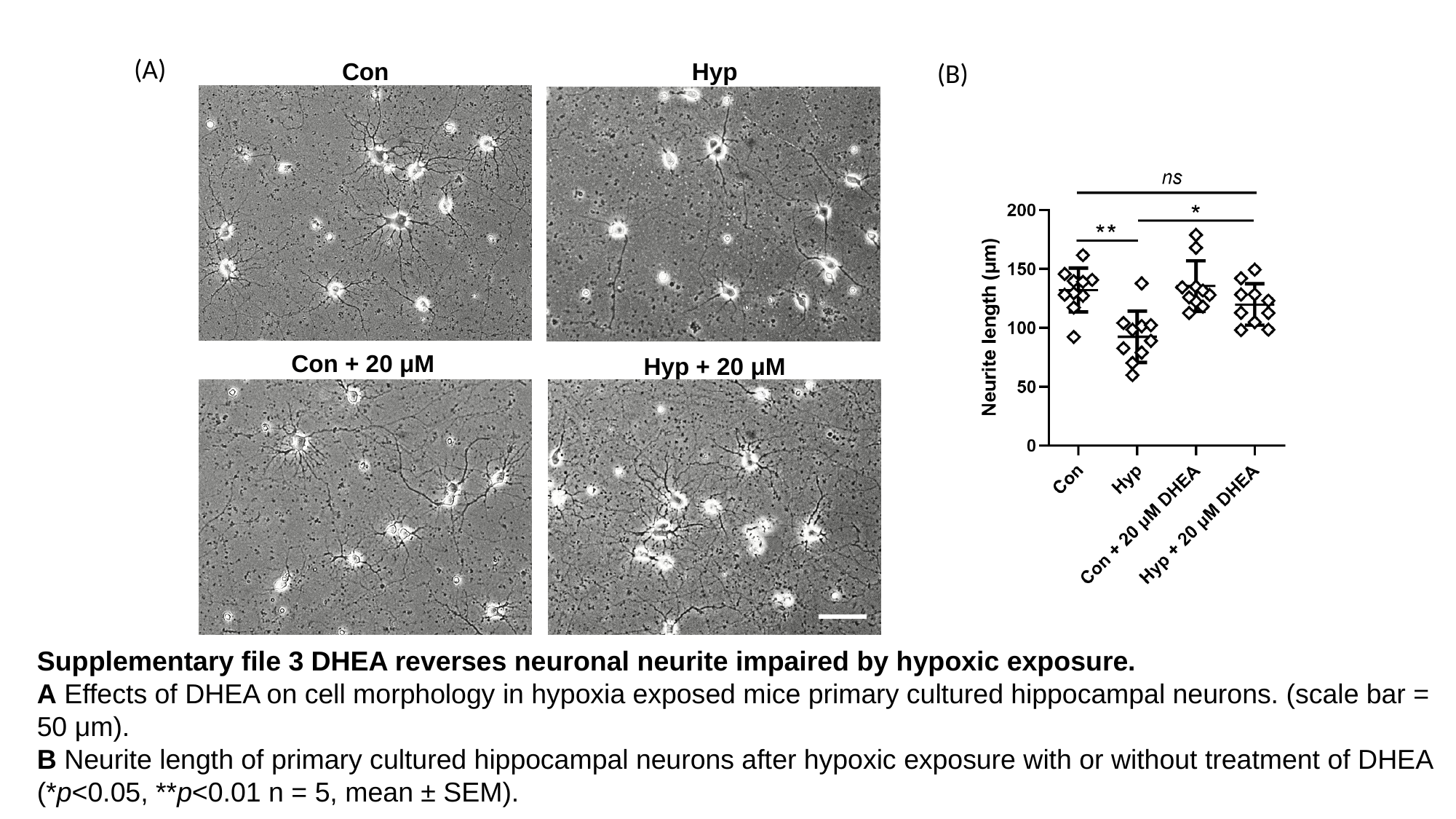

(A)
Hyp
Con
Con + 20 μM DHEA
Hyp + 20 μM DHEA
(B)
Supplementary file 3 DHEA reverses neuronal neurite impaired by hypoxic exposure.
A Effects of DHEA on cell morphology in hypoxia exposed mice primary cultured hippocampal neurons. (scale bar = 50 μm).
B Neurite length of primary cultured hippocampal neurons after hypoxic exposure with or without treatment of DHEA (*p<0.05, **p<0.01 n = 5, mean ± SEM).
